# Treatment of Vesical Atony by Ergotine Injections

**Published:** 1879-10

**Authors:** 


### TREATMENT OF VESICAL ATONY BY ERGOTINE INJECTIONS.

In three cases of vesical atony observed in old patients, Professor Langenbeck has obtained the best results from hypodermic injections of ergotine. Immediately after the injection the contractile power of the bladder was augmented and the patients micturated more abundantly. At the end of some days the bladder emptied itself almost entirely. In an old man, sixty-two years of age, who three or four times a day expelled about thirty grammes of urine, when his bladder contained more than half a litre, the very same day on which an hypodermic injection of twelve centigrammes of Benjeau's ergotine was employed, micturition was accomplished most satisfactorily; the prostate soon diminished in volume, and after four injections the cure was complete.—*L'Union Medicale.*

